# Intraosseous access can be taught to medical students using the four-step approach

**DOI:** 10.1186/s12909-017-0882-7

**Published:** 2017-03-02

**Authors:** Monika Afzali, Ask Daffy Kvisselgaard, Tobias Stenbjerg Lyngeraa, Sandra Viggers

**Affiliations:** 1 Department of Anaesthesiology, University Hospital of Copenhagen, Herlev, Denmark; 2 Cochrane Anaesthesia, Critical and Emergency Care, The Cochrane Collaboration, Herlev, Denmark; 3 Students’ Society of Anaesthesiology & Traumatology, Faculty of Health and Medical Sciences, University of Copenhagen, Herlev, Denmark; 4 Department of Anaesthesiology, Nordsjælland Hospital, Herlev, Denmark; 5 Copenhagen Academy for Medical Education and Simulation, Capital Region of Denmark, Herlev, Denmark

**Keywords:** Intraosseous access, Vascular access, Medical students, Medical education, Anaesthesiology, Traumatology, Emergency medicine, Resuscitation, OSCE and checklist validity

## Abstract

**Background:**

The intraosseous (IO) access is an alternative route for vascular access when peripheral intravascular catheterization cannot be obtained. In Denmark the IO access is reported as infrequently trained and used. The aim of this pilot study was to investigate if medical students can obtain competencies in IO access when taught by a modified Walker and Peyton’s four-step approach.

**Methods:**

Nineteen students attended a human cadaver course in emergency procedures. A lecture was followed by a workshop. Fifteen students were presented with a case where IO access was indicated and their performance was evaluated by an objective structured clinical examination (OSCE) and rated using a weighted checklist. To evaluate the validity of the checklist, three raters rated performance and Cohen’s kappa was performed to assess inter-rater reliability (IRR). To examine the strength of the overall IRR, Randolph’s free-marginal multi rater kappa was used.

**Results:**

A maximum score of 15 points was obtained by nine (60%) of the participants and two participants (13%) scored 13 points with all three raters. Only one participant failed more than one item on the checklist. The expert rater rated lower with a mean score of 14.2 versus the non-expert raters with mean 14.6 and 14.3. The overall IRR calculated with Randolph’s free-marginal multi rater kappa was 0.71.

**Conclusion:**

The essentials of the IO access procedure can be taught to medical students using a modified version of the Walker and Peyton’s four-step approach and the checklist used was found reliable.

## Background

Life-threatening emergencies with intravascular volume depletion, shock or cardiac arrest make peripheral venous access difficult. To ensure adequate resuscitation, alternative methods to obtain access to the venous system can be necessary.

Central venous catheter (CVC) and intraosseous (IO) cannulation can be used as an alternative to obtain vascular access in critically ill patients. These methods are suitable for the administration of fluids, blood products, and medications [1, 2]. Insertion of a CVC requires a high level of competence and can be time-consuming and difficult during resuscitation [3, 4]. The IO access has been shown to be easier to perform than peripheral venous catheter (PVC) insertion [5]. The IO needle insertion has few associated risks, a high operator satisfaction and a high rate of success even for the inexperienced clinician [3, 6–10]. Furthermore, substances injected by the IO route achieve adequate plasma concentrations in a time comparable with vascular access [11–14].

The American Heart Association (AHA) and the European Resuscitation Council (ERC) both recommend the use of IO access in cardiac arrest if PVC is not accessible [15, 16]. Recommendations from ERC in both paediatric and adult resuscitation are to establish an IO access if PVC cannot be achieved within one and two minutes respectively [15, 17]. There are no absolute contraindications for establishing the IO access as it is used on vital indication and complications such as infection and compartment syndrome are rare [3, 6–8, 18–20].

In Denmark a battery-powered IO driver (EZ-IO®) is the most commonly available device in the Danish emergency departments (EDs). A Danish study showed that in 2010 the IO device was available in 74% of the EDs. However, only 11% consistently established IO access on relevant indication and prior training in the establishment of IO access had not been provided in 47% of the EDs [21].

Procedural skills in Danish medical schools are traditionally taught during clinical rotations and in skill labs. Theoretical teaching or practical training in the use of the IO devices is however not a part of the curriculum. Most medical students and junior doctors have limited experience with the IO devices and a lack of introduction and training is a possible reason for the limited use in the Danish EDs.

The aim of this pilot study was to investigate if medical students can obtain competencies in IO needle insertion in human cadavers when taught by a modified Walker and Peyton’s four-step approach evaluated by an objective structured clinical examination (OSCE).

## Methods

In November 2013 Students’ Society of Anaesthesiology and Traumatology (SATS) at the University of Copenhagen (UCPH) conducted a four-hour human cadaver course in emergency procedures. Nineteen medical students, all members of SATS, participated [22]. The emergency procedures taught included emergency cricothyroidotomy, decompression of tension pneumothorax, chest tube insertion, and IO needle insertion. To participate in the course, students were required to have passed the 3rd semester of medical school studies at UCPH, which includes the anatomy dissection course. Pre-course material including a scientific paper describing the IO access technique, indications, and complications was sent to the participants prior to the course [19].

At the beginning of the course there was a 30-min lecture regarding the indications and contraindications of the IO access presented by a registrar in anaesthesiology. This session was followed by a one-hour workshop. The workshop was carried out using a modified version of Walker and Peyton’s four-step approach as the teaching method [23, 24]. The modified four-step approach consists of four phases. An initial “Demonstration” phase where participants observe while the instructor performs an IO needle insertion, just as it would be performed in real life and real time. Secondly, a “Deconstruction” phase where the instructor performs the task slowly while verbally breaking the performance down into simple steps (Table 1). The third phase is the “Formulation” where the instructor performs the task while being “talked through it” by the participants step-by-step. Finally, in the fourth “Performance” phase the participants perform the task themselves while the instructor gives the participants immediate feedback. The participants were allowed to repeat step four and practice the skill until they felt confident with the procedure.

**Table 1:** Important steps for achieving intraosseous access

Task No.	Procedure Description
1	Identify insertion site. Two fingers down from the tibial tuberosity and one finger medially.
2	Prepare the insertion site with antiseptic swab.
3	Attach needle to the driver and prime the connector.
4	Align needle almost perpendicular to the bone with 10–15° angulation away from knee joint.
5	Insert the needle through the skin without drilling until firm contact with bone. Verify correct needle size by visual inspection of markings on needle above skin level.
6	Proceed with insertion by squeezing the trigger and applying gentle pressure while drilling continuously until loss or decrease in resistance.
7	Stop drilling when feeling loss of resistance and steady the needle with one hand while removing upper part of needle and attaching connector.
8	Secure needle and connector properly using stabilizer.
9	Verify correct placement by aspiration of blood and administration a saline flush without signs of extravasation.

**Fig. 1:**
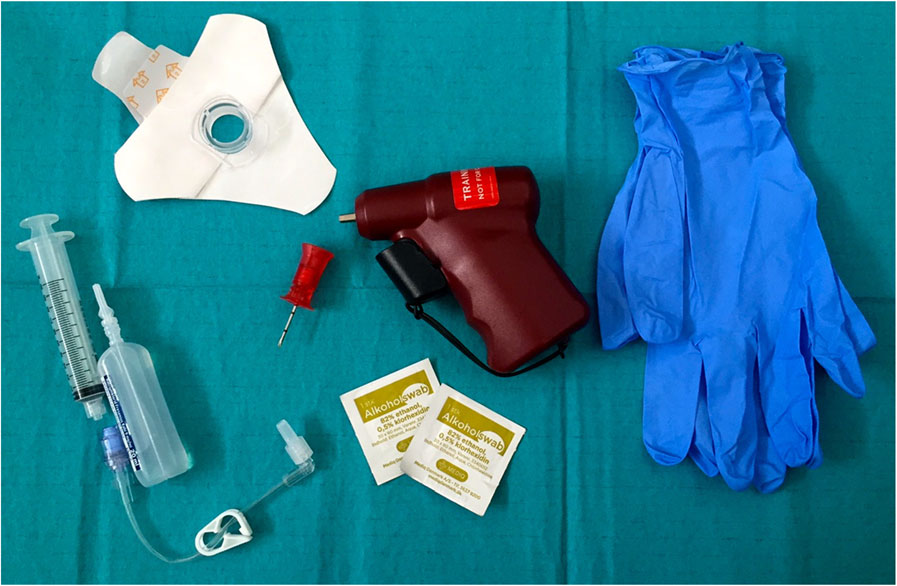
The necessary equipment for the IO procedure

After the course, the participants’ competencies in IO needle insertion were evaluated. An OSCE was chosen as the test modality for skill evaluation [25]. Participants were presented with a case where IO access was indicated (Table 2). All necessary equipment was made available and the proximal tibia of the human cadaver was exposed (Fig. 1). For the OSCE, an earlier validated weighted checklist for manual IO cannulation was modified by the authors (SV and TL) [26, 27]. They identified nine steps as important for successful achievement of an IO access with a battery-powered IO driver (Table 1). These steps inspired to a new checklist (Table 3). Weighted points were awarded for the task being performed correctly otherwise zero points were assigned. The checklist allowed a maximum score of 15 points (Table 3).

**Table 2:** A case where IO access is indicated

“You have been called to the emergency room where a patient is unconscious. During the primary survey you establish the need for a vascular access to initiate resuscitation with fluids and medications. Failure to gain access with a peripheral venous catheter calls for the use of the intraosseous device. The equipment needed is placed on the table in front of you. Feel free to talk out loud while performing the procedure, however this is not a requirement.”

**Table 3:** Checklist for the intraosseous access workshop

Topical antiseptic	
Not used	0
Used on the puncture site	1
Gloves	
Not used	0
Gloves used	1
Insertion technique	
Skin penetration with needle while drilling	0
Skin penetration with needle placed on the machine without drilling	1
Further insertion of IO needle with discontinued drilling until loss of resistance	0
Further insertion of IO needle with continuous drilling until loss of resistance	2
Fluid aspiration from marrow cavity with an empty syringe	
Absence of aspiration	0
Aspiration of fluid	2
Infusion of 0,9% NaCl	
Absence of infusion of 0,9% NaCl	0
Infusion of 0,9% NaCl	2
Securing the line	
No further securing of IO line	0
Use of dedicated stabilizer and/or other fixation to secure the line	1
Location of the puncture site (observed after insertion)	
Outside the puncture site or mobile needle	0
On the puncture site +/− 0,5 cm	3
Angle of insertion (observed after insertion)	
Oblique insertion	0
Perpendicular insertion +/− 10°	2
Total Score	
I.O access functioning	Yes_____
	No______

As the participants performed the procedure, the three authors AK (rater one), MA (rater two) and TL (rater three) independently rated the performance using the weighted checklist. Consequently, each participant had three checklists assessing their performance. Rater one (R1) was a second year medical student, rater two (R2) was a sixth year medical student and rater three (R3) was a registrar in anaesthesiology. The two raters R1 and R2 were considered non-expert raters and R3 was considered the expert rater.

Statistics were performed using IBM SPSS statistics for MAC version 22.0. Armok, NY: IBM corp. Cohen’s *kappa* was performed to assess the inter-rater reliability (IRR) between the non-expert raters and the expert rater [27, 28]. To examine the strength of the overall IRR, Randolph’s free-marginal multi rater *kappa* was used to evaluate the validity of the checklist [28–30].

## Results

Out of the 19 students who participated in the course 15 (79%) participated in the final OSCE. Nine out of the 15 participants (60%) obtained a total score of the maximum 15 points and two participants (13%) scored 13 points with all three raters. For the remaining four participants there was non-agreement among the raters with a participant median score of 14.5 (range 10–15).

The two participants who scored 13 points with all three raters both failed one assessment point on the checklist. The failed assessment points were respectively; “Infusion of 0.9% NaCl” and “Fluid aspiration from marrow cavity with an empty syringe”.

In four participants there was non-agreement among the raters. Three out of four had non-agreement on a single assessment point “insertion technique (drilling)”, “securing the line” or “location of puncture site”, respectively. Only one participant failed more than one item on the checklist and led to non-agreement on two assessment points; “insertion technique (drilling)” and “location of puncture site”. On both assessment points for this participant, all raters had made comments on the checklists related to the execution of the tasks.

In two out of the five incidents of non-agreement on assessment points, there was discrepancy between expert and non-expert raters. The expert rater R3 rated lower with a mean score of 14.2 (CI: ±0.72 |13.48;14.92|) versus the non-expert raters with R1 mean score 14.6 (CI: ±0.41 |14.19;15.00|) and R2 14.3. (CI: ±0.65 |13.65;14.95|) The IRR between the non-expert rater R1 and the expert rater R3 was 0.66 showing a substantial level of agreement and IRR between R2 and R3 was 0.59 showing a moderate level of agreement. The level of agreement between the two non-expert raters R1 and R2 was 0.56 – also showing a moderate level of agreement. The overall IRR calculated with Randolph's free-marginal multi rater *kappa* was 0.71 indicating a substantial level of agreement between the three raters.

## Discussion

The essentials of the IO access procedure can be taught to medical students as part of a four-hour human cadaver course using a modified version of the Walker and Peyton’s four-step approach. A modified version of a previously validated checklist to assess the performances of 15 medical students in gaining IO access was used. This resulted in 60% (*n* = 9) of the participants obtaining the highest attainable points with all three raters. The checklist for assessing and evaluating performance of achieving IO access in human cadavers was found reliable with a free-marginal kappa value of 0.71. This implied a substantial overall IRR. However, the interpretation of a multi-rater *kappa* must be made with caution in small study populations. The IRR between the non-expert and expert raters showed that peer-assessment might be problematic compared to assessment performed by a more experienced clinician. Raters jointly observing and discussing a case of good performance and poor performance and agreeing on a score may ensure more uniform rating of performance in the future. Training of raters beforehand may also decrease the likelihood of guessing and further increase IRR for both non-expert and expert raters [29].

The four-step teaching approach by Walker and Peyton is a well-established method in teaching surgical procedures and PVC and its implementation for procedural skill teaching is recommended [23, 24, 31]. The approach is developed for 1:1 teaching but students also welcome it for small group training [24]. The use of this teaching method at a cadaver course may increase focus on making use of this teaching method when teaching medical students and other novice learners practical skills outside the simulation centre or skill lab. However, when evaluating the modified four-step approach used in this study, one must be aware that the teaching outcome could have been increased because of a final OSCE, as testing has been shown to increase skill learning [32].

No minimal score was set as a value for passing or failing the checklist in this study. The previously validated checklist for manual IO cannulation used a score above 15 out of 20 possible points (>75%) as sufficient for passing - even if some of the highest weighted points on the checklist were failed [24]. If this were to be applied in our study a score of 12 points would be sufficient to pass. Using this as a pass value retrospectively all students in this study would have passed based on the median scores, whereas one would have failed based on the expert rater’s score. Deciding on a minimal score and the use of essential points that are not to be failed should be implemented to ensure adequate power of the checklist in distinguishing between failed and successful IO-placement. Furthermore, addition of time to IO insertion and time spent on insertion should be included if the checklist is to be used for assessment of skill proficiency in a live patient population.

Failure to achieve IO access is related to technical difficulties or failure to locate correct insertion site [18]. This underlines the importance of training and assessment of those who will perform the procedure. In Denmark IO access is taught at courses such as European Paediatric Life Support and Advanced Trauma Life Support but the use in the clinical setting remains infrequent [19]. Teaching students how to obtain an IO access during medical school may result in more frequent use in the future, increase competence by allowing time for spaced repetition of the skill, and improve initial resuscitation attempts and consequently patient outcome.

Development of formal training in the use of IO devices may help to ensure proper application of IO access when indicated according to AHA and ERC guidelines.

The proximal tibia was chosen as the insertion site for both the workshop and the final OSCE and a battery-powered IO driver device was used. Studies indicate that this device is superior to other devices as it is easy to use and holds a high success rate of insertion among users [11, 33].

The proximal tibia and the humeral head are often the preferred site of IO needle insertion [5, 8, 34]. A randomized study on 182 patients during out-of-hospital cardiac arrest shows that achieving IO access at the proximal tibia is faster and more often successful compared to either PVC insertion or IO placement in the humeral head [5]. This might be due to on-going activity around the humerus during resuscitation increasing rates of dislodgement [5, 34]. Placement in the proximal tibia rather than the humeral head is also easier on human cadavers due to tissue preservation and comparison of flow rates shows no significant difference [8].

### Limitations

As the OSCE was performed immediately after the workshop session the retention of the skill taught was not tested and the possibility that the test itself enhanced the learning outcome is possible [35]. Furthermore a total of four of the participants chose not to participate in the final OSCE. It was not investigated further why these participants declined the OSCE. However, this could be because they did not feel adequately prepared for being tested and their exclusion from the tested group may affect the results positively.

No demographical data about the participants were collected, however all participants had passed anatomy class, as this was a pre-requisite for participation. We assumed that participants did not have any experience with IO insertion techniques from training or clinical setting, as IO access is not part of clinical skills curriculum at UCPH medical school. Their theoretical knowledge was therefore not tested pre- or post-course as it was expected that the participants’ knowledge would increase no matter what teaching approach was being used.

## Conclusion

This study demonstrates that within a four-hour time frame the fundamentals of achieving an IO access can be taught to medical students through a human cadaver course using Walker and Peyton’s’ four-step approach as the teaching method. Assessment using a checklist can be a reliable way to assess the students but for future implementation we recommend raters with prior education in the checklist, a crossover design as well as testing different learning modalities against each other. To ensure retention of the gained knowledge we suggest repetitive training in achieving IO access to be integrated as a part of medical school’s clinical skills curriculum to ensure the highest standard of care in emergency situations and to maximize skill retention.

## Abbreviations

AHA: American Heart Association
CVC: Central venous catheter
ED: Emergency department
ERC: European Resuscitation Council
IO: Intraosseous
IRR: Inter-rater-reliability
OSCE: Objective structured clinical examination
PVC: Peripheral venous catheter
R1: Rater one
R2: Rater two
R3: Rater three
UCPH: University of Copenhagen
